# Rethinking the Meaning of Cloud Computing for Health Care: A Taxonomic Perspective and Future Research Directions

**DOI:** 10.2196/10041

**Published:** 2018-07-11

**Authors:** Fangjian Gao, Scott Thiebes, Ali Sunyaev

**Affiliations:** ^1^ Department of Information Systems University of Cologne Cologne Germany; ^2^ Department of Economics and Management Karlsruhe Institute of Technology Karlsruhe Germany

**Keywords:** cloud computing, taxonomy, health IT innovation

## Abstract

**Background:**

Cloud computing is an innovative paradigm that provides users with on-demand access to a shared pool of configurable computing resources such as servers, storage, and applications. Researchers claim that information technology (IT) services delivered via the cloud computing paradigm (ie, cloud computing services) provide major benefits for health care. However, due to a mismatch between our conceptual understanding of cloud computing for health care and the actual phenomenon in practice, the meaningful use of it for the health care industry cannot always be ensured. Although some studies have tried to conceptualize cloud computing or interpret this phenomenon for health care settings, they have mainly relied on its interpretation in a common context or have been heavily based on a general understanding of traditional health IT artifacts, leading to an insufficient or unspecific conceptual understanding of cloud computing for health care.

**Objective:**

We aim to generate insights into the concept of cloud computing for health IT research. We propose a taxonomy that can serve as a fundamental mechanism for organizing knowledge about cloud computing services in health care organizations to gain a deepened, specific understanding of cloud computing in health care. With the taxonomy, we focus on conceptualizing the relevant properties of cloud computing for service delivery to health care organizations and highlighting their specific meanings for health care.

**Methods:**

We employed a 2-stage approach in developing a taxonomy of cloud computing services for health care organizations. We conducted a structured literature review and 24 semistructured expert interviews in stage 1, drawing on data from theory and practice. In stage 2, we applied a systematic approach and relied on data from stage 1 to develop and evaluate the taxonomy using 14 iterations.

**Results:**

Our taxonomy is composed of 8 dimensions and 28 characteristics that are relevant for cloud computing services in health care organizations. By applying the taxonomy to classify existing cloud computing services identified from the literature and expert interviews, which also serves as a part of the taxonomy, we identified 7 specificities of cloud computing in health care. These specificities challenge what we have learned about cloud computing in general contexts or in traditional health IT from the previous literature. The summarized specificities suggest research opportunities and exemplary research questions for future health IT research on cloud computing.

**Conclusions:**

By relying on perspectives from a taxonomy for cloud computing services for health care organizations, this study provides a solid conceptual cornerstone for cloud computing in health care. Moreover, the identified specificities of cloud computing and the related future research opportunities will serve as a valuable roadmap to facilitate more research into cloud computing in health care.

## Introduction

### Background and Objective

Cloud computing (CC) is an innovative paradigm that provides users with on-demand access to a shared pool of configurable computing resources such as servers, storage, and applications [1]. CC possesses unique features (ie, on-demand self-service, broad network access, resource pooling, rapid elasticity, and measured services) that are argued to enhance traditional in-house health information technology (IT) approaches in health care organizations (eg, hospitals and clinics). Researchers claim that IT services delivered via the CC paradigm provide major benefits for health care, including improved flexibility in the use of IT resources [2], high availability of IT infrastructure to address ever-changing health IT demands [3], and low upfront investments and IT maintenance costs for the use of health IT [4]. Surprisingly, the benefits promised by using CC often do not hold in practice: it has, for example, been reported that the use of cloud computing services (CCSs) is tied to implementation and preparation activities that impede the flexibility of CC [5], the promised high availability of cloud-based IT infrastructures also cannot always be ensured (eg, sometimes the maximal attainable IT resources are strictly predefined) [6], and the use of CCSs is not guaranteed to yield the expected economic advantages for users in health care (eg, due to unexpected high upfront costs) [7,8]. There is therefore a mismatch between our conceptual understanding and the accepted meaning of CC for health care (ie, the value and/or consequences of using CC) in practice. Such a mismatch not only hampers the meaningful use of CC in the health care industry (ie, CC should provide constructive support) [9] but also could lead to countereffects for health care. As reported in a recent case, performance of an electronic health record system enabled by CC in a United Kingdom hospital diverged from initial expectations and led to countereffects, resulting in a £200 million (US $262 million) project failure and the hospital’s inability to deliver key services on a large scale [10,11].

Although the topic of CC in health care has been widely discussed in the literature, existing publications mainly focus on development of single CC applications or platforms in health care [12-16] and development of security mechanisms for the use of CC [17-21]. Although some studies have tried to conceptualize CC or interpret this phenomenon for health care settings [4,22,23], they are heavily based on a general understanding of traditional health IT artifacts or mainly rely on the interpretation of CC in a common context, which leads to an insufficient or unspecific conceptual understanding of CC for health care. CC is an IT innovation for the health care industry that differs from traditional health IT approaches; in addition, when conceptualizing the topic of CC in health care, it is essential to seriously consider the health care context. The health care industry is markedly different from the commonly understood context and interpretation of CC [24]. Thus, this more general CC context is not necessarily adequate for health care. To this end, past research suggests that a nonspecific grasp of the CC concept in research and practice, irrespective of the intricacies of the health care sector, might be a major reason for why few successful implementations of CCSs in health care exist [25].

In this research, we rethink the meaning of CC for health care. By relying on existing CCSs in practice, we aim at generating insights into this phenomenon for health IT research. Our research focuses on the following research questions (RQs):

RQ1: What are the relevant properties of CC for service delivery to health care?

RQ2: What are the specific meanings of these properties for health care?

To address the research questions, we drew on data from a structured literature review and 24 expert interviews to develop a taxonomy of CCSs for health care organizations. Taxonomies are a form of classification [26] that are widely used to understand IT concepts in health care [27,28]. We expect to use this taxonomy to organize existing knowledge about CC in health care to fulfill our research purpose. In particular, we relied on the taxonomy to understand CC’s key service delivery properties for health care organizations (RQ1) and thereby conceptualized CC for health care settings. By classifying 50 CCSs for health care organizations that we identified from both the literature and interviews using the taxonomy, we derived specificities of CC for health care (RQ2) that subverted and, therefore, challenged our understanding of CC in a common context or from a traditional health IT perspective. Our study conceptualizes CC specifically for health care. More importantly, we derived concrete research directions based on our conceptualization of CC to facilitate research on CC in health care.

### Cloud Computing Knowledge in Health Care

CC is an innovation for health care organizations. In the health care industry, 3 types of innovations can be observed: (1) innovation focusing on the manner in which consumers access health care and fund the related services; (2) innovation applying technology to improve products, services, or care; and (3) innovation generating new business models [29]. CC is an innovation of applying (information) technology in health care organizations (type 2) that is in sharp contrast to traditional health IT approaches. CC provides 3 different service models—software as a service (SaaS), platform as a service (PaaS), and infrastructure as a service (IaaS)—all of which are Web-based [1]. CC can therefore deliver fundamental IT resources such as processing, storage (IaaS), and platforms together with programming languages, tools, and/or libraries that support users to develop and/or deploy software (PaaS). CC can also provide ready-to-use software applications (SaaS), which run on the cloud infrastructure, to health care organizations.

CC relies on different deployment models to provide IT services. First, in a public cloud, the infrastructure of CCSs is provided for open use by the general public. Second, the infrastructure of a private or community cloud is provisioned for the exclusive use by a single organization or a specific group of organizations, respectively. Third, a hybrid cloud is a combination of 2 or more of the aforementioned deployment models. Whereas public clouds exist off the premises of cloud users, private and community clouds may exist on or off premises.

Our research aimed at organizing knowledge about CC and conceptualizing CC in health care. We employed the concept of knowledge about innovations by Rogers [30] as a means to interpret the knowledge about CC in health care and guide the taxonomy development. We chose it because Rogers’ concept of knowledge is one of the few established concepts in research that can specify an IT artifact by observing it as an innovation, which is appropriate for CC as an innovation in health care. Moreover, Rogers’ knowledge about innovations serves as a basic concept in his diffusion of innovations theory. Although we did not specifically address issues regarding CC’s diffusion, we aimed for a specific understanding of an innovation (in health care), which is consistent with Rogers’ ultimate purpose for this concept in the diffusion of innovations theory.

According to Rogers, 3 different types of knowledge are relevant for an insightful understanding of an innovation: (1) awareness knowledge comprises information about the existence of an innovation, (2) how-to knowledge describes how the innovation can be applied, and (3) principle knowledge explains the approach in which an innovation works. In this research, we targeted how-to and principle knowledge to understand the term knowledge. This is because most are aware of the term “cloud computing” [31]. Our research focused on the properties of CCSs that describe how CC can be used in health care organizations (how-to knowledge) and the ways in which CCSs support health care organizations (principle knowledge).

## Methods

### Overview

**Figure 1:**
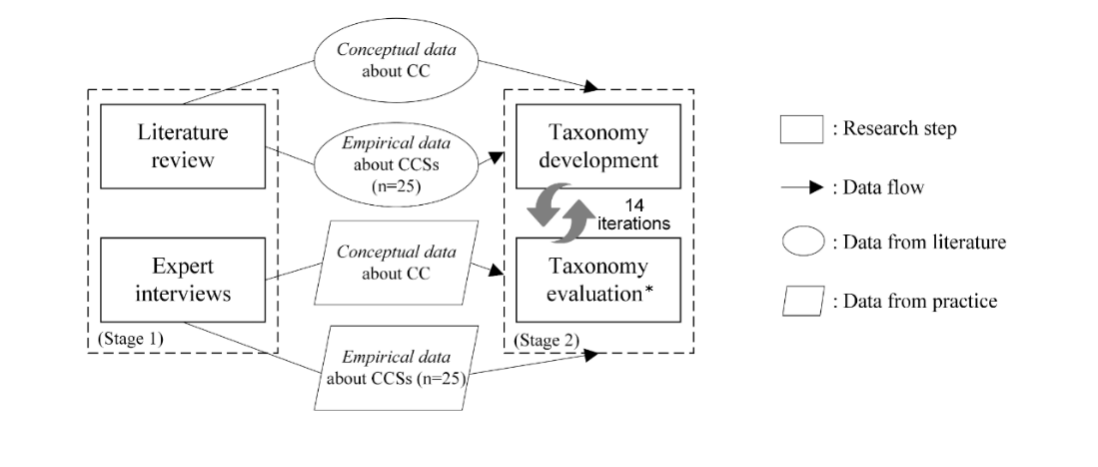
Research methods overview. Asterisk refers to taxonomy evaluation by means of the ending conditions. CC: cloud computing, CCS: cloud computing service.

We employed a 2-stage approach to develop a taxonomy of CCSs for health care organizations. As illustrated in Figure 1, we conducted a structured literature review and 24 semistructured expert interviews in stage 1, drawing on data from theory and practice. In stage 2, we employed the views of how-to and principle knowledge, applied the method used by Nickerson et al [32], and developed a taxonomy of CCSs for health care organizations. The taxonomy development method integrates the evaluation of the taxonomy into its development process such that no further a posteriori evaluation of the taxonomy was required.

### Literature Review

**Figure 2:**
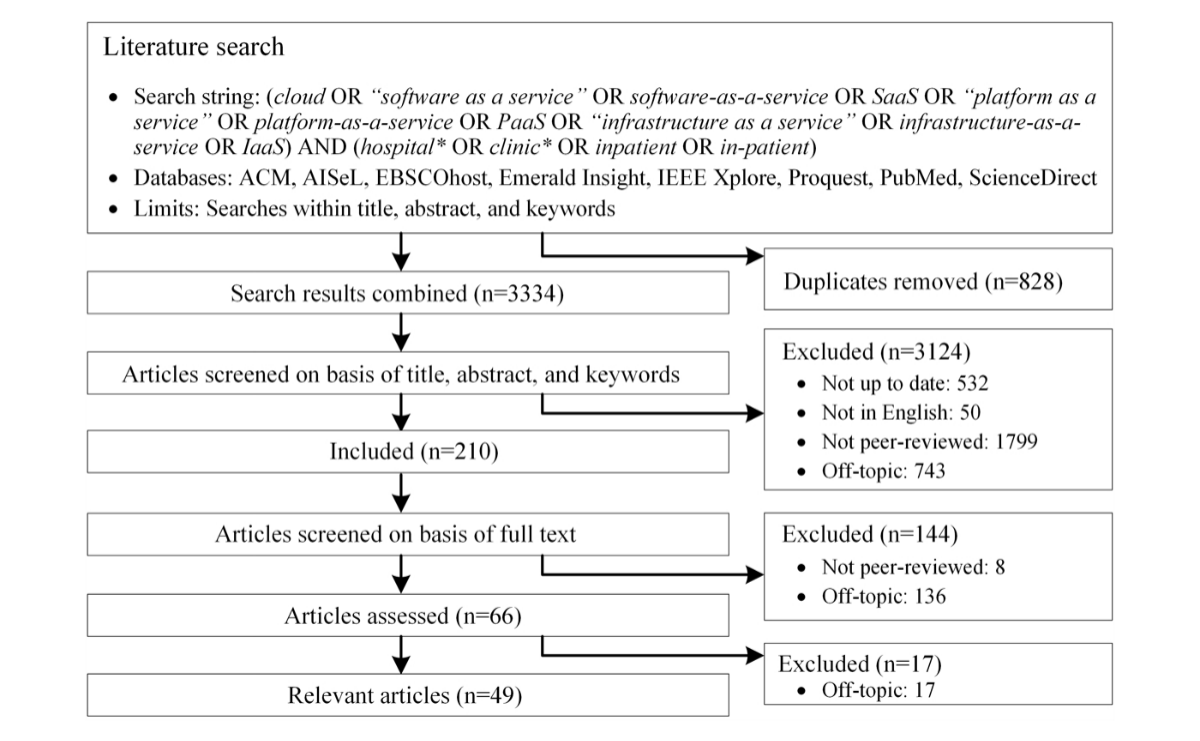
Flow diagram of inclusion/exclusion and literature analysis.

To obtain data for the development of our taxonomy, we followed the Preferred Reporting Items for Systematic Reviews and Meta-Analyses framework [33] and performed a review of the literature on CC in health care organizations. We searched literature databases to identify research articles addressing the topic of CC in health care organizations. Figure 2 presents a schematic of our approach, which includes the literature databases and the search string employed. It must be emphasized that we iteratively developed our search string. We tested broader keywords (eg, “eHealth,” “health IT”) but decided to employ more specific keywords that target health care organizations for the final search string because our taxonomy specifically focused on health care organizations. Moreover, we found that the broader keywords did not result in many additional relevant articles but increased noise, which diminished the quality of the literature review. We performed keyword, title, and abstract searches and ultimately full-text reviews. Next, 2 researchers independently screened the identified articles. The articles were first screened using keywords, titles, and abstracts and then using the full texts. We excluded articles that were not published within the last 10 years (not up to date: the term CC was not readily used until 2007), not in English, not peer-reviewed, or did not address the topic of CC in health care organizations (off-topic). A total of 66 articles remained after the screening.

Once the screening was complete, we analyzed the remaining articles and identified 17 additional articles that were off-topic but could not have been excluded without an in-depth full-text assessment. This process resulted in a final sample of 49 eligible articles that were assessed in detail. With the assessment, we aimed to understand the concept of CC in health care organization contexts from a research perspective. Moreover, we attempted to identify concrete CCSs for health care organizations in addition to their characteristics from the literature. Accordingly, we classified the literature into 2 categories: conceptual and empirical. The conceptual category covered articles providing general conceptual statements about CC in health care and articles proposing CCSs that have not been deployed in practice. The empirical category contained articles describing concrete CCSs for health care organizations. This occurred because the applied taxonomy development method employed both a deductive approach (development based on data from the conceptual category) and an inductive approach (development by observing objects that need to be classified, namely, data from the empirical category) [32]. Of the 49 eligible articles, 24 were classified as conceptual and 25 as empirical. Articles that describe general features of CC and apply them to concrete CCSs were classified as special cases of the empirical category. Two researchers separately analyzed the articles. Each relevant statement was extracted and converted into 1 or more pieces of code representing a property of CCSs for health care organizations. Codes created by both researchers were compared and aggregated resulting in a master list containing codes encapsulating the properties of CCSs. The master list covers codes from both the conceptual (ie, general conceptual understanding of CC) and empirical categories (ie, concrete CCSs and their properties). It must be emphasized that 25 concrete CCSs for health care organizations were identified from the literature. A description of these CCSs can be found in Multimedia Appendix 1.

### Expert Interviews

To gather knowledge that could inform the development of the taxonomy from practice, we conducted 24 semistructured expert interviews, as listed in Table 1. We applied a purposeful sampling strategy that focused on selecting individuals who are especially knowledgeable about a phenomenon of interest to recruit interviewees [34]. We included only experts who were engaged in IT activities in health care organizations and who had used, provided, or knew about concrete CCSs for health care organizations. After 24 interviews, we reached data saturation and stopped recruiting additional interviewees. The first 12 interviewees listed in Table 1 focus on the Chinese health care cloud market, and the rest focus on the German market. We selected these countries because they are the main cloud players in Asia and Western Europe, which are among the regions with the highest market share in the overall [35] and the health care cloud markets [36]. Moreover, the cloud markets in China and Germany are complementary to each other: whereas CCSs for health care organizations in Germany are restricted to European cloud providers due to data protection regulations by the European Union, CCSs in China rely on large health IT players (eg, IBM, Cisco, and Microsoft) mainly from the United States supplemented by Chinese domestic providers [37]. Thus, we were able to gain insights into knowledge about CC in health care from a wide spectrum of practices. The interviewees came from 18 different organizations and had an average of 15 years of work experience.

**Table 1:** Overview of interviewees.

ID	Job title	Experience in health IT^a^ (years)	Work organization
i01	Chief information officer	8	General hospital in China
i02	Chief of information center	18	General hospital in China
i03	Project manager	12	International health IT provider
i04	Staff of new media department	6	Specialized hospital in China
i05	Chief of IT department	15	District clinic in China
i06	Chief executive officer	16	Chinese health IT provider for dental clinics
i07	Senior IT staff	12	General hospital in China
i08	IT supervisor	17	Chinese governmental organization for the strategic development of public hospitals
i09	Chief of information center	11	General hospital in China
i10	Senior IT staff	9	General hospital in China
i11	Vice director	12	District hospital in China
i12	Head of IT	6	General hospital in China
i13	Chief marketing officer	33	Health IT provider for the German market
i14	Staff of research and development department	30	Health IT provider for the German market
i15	Head of IT applications	20	University clinic in Germany
i16	Technology officer	10	Health IT provider for the German market
i17	Head of IT development	6	German local health IT provider
i18	Health IT developer	6	German local health IT provider
i19	Senior manager	19	German local health IT provider
i20	Head of IT	17	University clinic in Germany
i21	IT staff	10	University clinic in Germany
i22	IT team leader	19	University clinic in Germany
i23	Chief information officer	12	District hospital in Germany
i24	Head of IT infrastructure	31	University clinic in Germany

^a^IT: information technology.

Our interview guide was structured into 3 topics, as shown in Multimedia Appendix 2. Topic 1 addressed the interviewee’s organization, work activities, and professional experience. Topic 2 focused on the interviewee’s (conceptual) understanding of CC in health care. In topic 3, interviewees were asked to enumerate and describe all concrete CCSs in health care organizations with which they were familiar. The interviews lasted between 30 and 90 minutes, with an average of 51.33 minutes. All interviews were audio recorded and transcribed afterwards.

Two researchers separately analyzed the transcripts. For the same reasons as in the literature analysis, the interview analysis focused on not only the conceptual understanding of CC in health care but also concrete examples of CCSs, including their properties. Thus, we classified the interview data obtained from topic 2 of the interview guide in the conceptual category, whereas the interview data obtained from topic 3 fell into the empirical category. Both researchers employed the same coding technique used in the literature analysis to analyze the interview data. Consequently, we obtained a list of codes representing a conceptual view of CC in health care for the conceptual category and a list of codes representing properties of concrete CCSs in health care organizations for the empirical category. In total, 25 CCSs for health care organizations were identified from the interviews, which are presented together with the 25 CCSs identified from the literature in Multimedia Appendix 1.

### Taxonomy Development

For the taxonomy development, we chose the method proposed by Nickerson et al [32], which provides a systematic taxonomy development approach for IT objects and is well acknowledged in the domain of health IT [38,39]. According to Nickerson et al [32], a taxonomy is a set of dimensions in which each dimension consists of more than 1 characteristic. In taxonomy development, several iterations are used to determine dimensions and characteristics. After each iteration, predefined ending conditions are employed to evaluate the taxonomy: if not all ending conditions can be fulfilled, the taxonomy development continues with the next iteration. In each iteration, researchers can choose between an inductive and deductive approach. A deductive approach is based on theoretical knowledge about the objects that need to be classified; an inductive approach is based on observing and analyzing a sample of the objects. For the deductive approach, we applied all data about CC from the conceptual category (see Figure 1). For the inductive approach, we employed data from the empirical category for all 50 identified CCSs in health care organizations.

Before developing a taxonomy, researchers must define a meta-characteristic and ending conditions. The meta-characteristic guides the choice of dimensions and characteristics in the taxonomy. As a result, each dimension or characteristic of the taxonomy is a logical consequence of the meta-characteristic. Our taxonomy builds on 2 relevant knowledge types of CCSs to define the meta-characteristic: how-to and principle knowledge. We defined “service delivery properties of CCSs for health care organizations” as our meta-characteristic that covers how CCSs can be used by health care organizations (how-to knowledge) and describes the approaches in which CCSs support them (principle knowledge). Both knowledge types serve as the conceptual orientation of the taxonomy as a whole. For the ending conditions, we adopted all of the objective and subjective ending conditions from Nickerson et al [32]. The subjective ending conditions also serve as criteria to evaluate the sufficiency of the taxonomy.

For each iteration, we randomly chose a developmental approach (ie, inductive or deductive). Based on the chosen approach, we randomly selected data from our data pool accordingly (ie, understanding of CC from the conceptual category for a deductive approach and concrete CCSs and their properties from the empirical category for an inductive approach). The amount of data was adjusted such that each iteration could be performed in a reasonable time frame (45 to 60 minutes).

For an iteration using the deductive approach, we first examined codes about CC to identify and summarize new characteristics and/or dimensions. We determined whether each potential new characteristic or dimension derived from a code could be considered a logical consequence of the meta-characteristic and whether there was a concrete CCS in our empirical category that could be classified into this characteristic/dimension. If both criteria were fulfilled, the new characteristic/dimension was added to the existing taxonomy. For an iteration using the inductive approach, we first examined and compared the properties of the selected CCSs from the empirical category. We attempted to derive common characteristics of the chosen CCSs by comparing their codes. If the identified characteristics were new, we attempted to assign them to existing dimensions (as characteristics) if possible. Otherwise, we grouped the characteristics, inspected their conformity with the meta-characteristic, and defined them as new dimensions for the taxonomy, if necessary. After each iteration, we applied the predefined ending conditions to evaluate our taxonomy. For an inductive approach, we additionally classified all CCSs that were analyzed using the (preliminary) taxonomy, as required by Nickerson et al [32]. After 14 iterations, we met all ending conditions and thus stopped the taxonomy development. Multimedia Appendix 3 summarizes these iterations and the data we applied to each. Because all identified CCSs for health care organizations (n=50) were analyzed in our research (ie, an objective ending condition), these CCSs were classified by the taxonomy. The final classification result serves as a part of the taxonomy.

## Results

### Dimensions and Characteristics

Our taxonomy of CCSs for health care organizations is composed of 8 dimensions and 28 characteristics (see Table 2 for overview). The first 4 dimensions (service form, deployment model, targeted cloud advantage, and timeliness) represent principle knowledge, which is related to the inherent mechanisms and principles of a CCS and describes the approaches in which CC supports health care organizations. The remaining 4 dimensions address concrete methods to implement (ie, how to use) CCSs for health care and represent how-to knowledge.

The service form and deployment model dimensions are consistent with the service and deployment models of CC, respectively [1]. They clarify the most basic operational principles of CCSs for health care organizations, which relate to principle knowledge. The dimension service form contains 3 characteristics: infrastructure, platform, and software, which refer to IaaS, PaaS, and SaaS of CC, respectively. The deployment model dimension indicates whether CCSs are deployed using a public, community, or private cloud. Because a hybrid cloud is, by definition, composed of 2 or more of the aforementioned deployment models, we do not define hybrid as an independent characteristic of the deployment model. Instead, our taxonomy represents a CCS with a hybrid deployment model by using 2 or more of the characteristics defined above.

The targeted cloud advantage dimension describes the concrete cloud properties from which a health care organization can benefit. This dimension highlights the effects of using CCSs and is also considered a type of principle knowledge. Scalability refers to the advantage of a CCS that extends its IT resources (eg, storage, processing, and memory) to overcome a health care organization’s IT resource scarcity or support resource-intensive tasks. Elasticity represents a CCS’s capability to dynamically allocate available resources based on users’ demands and thus optimize resource use for all users. Ubiquity indicates that users can access the CCS from any location. Cost efficiency emphasizes the cost advantage brought by CCSs. Shareability refers to the ability of CCSs to enable the efficient exchange and sharing of data between different users, whereas interoperability denotes the ability of a CCS to smoothly integrate and operate with disparate systems and machines. Security allows health care organizations to take advantage of cloud providers’ advanced data security mechanisms or technologies.

Timeliness assesses how quickly CC is able to deliver services and related data to health care organizations (real time vs not real time) and thus relates to principle knowledge. We define a CCS as real time if it is ready to process or transfer data at any time, such that the computational results and requested data are immediately available.

**Table 2:** Taxonomy of cloud computing services for health care organizations.

Dimension			Characteristics
**Principle knowledge**			
	Service form	Software, platform, infrastructure	
	Deployment model	Public, private, community	
	Targeted cloud advantage	Scalability, elasticity, ubiquity, cost efficiency, shareability, interoperability, security	
	Timeliness	Real time, not real time	
**How-to knowledge**			
	Supported task	Clinical, administrative, strategy, research	
	User	Patient, medical staff, family member	
	Service delivery device	Independent, adapted, specialized	
	Patient data involvement	Internal, external, no involvement	

The supported task dimension specifies the areas in which health care organizations use CCSs. This dimension highlights the manner in which CC supports health care and is deemed a type of how-to knowledge. Supported task includes 4 characteristics: clinical, administrative, strategic, and research. Clinical refers to medical activities in health care organizations that are directly associated with patient diagnosis and treatment. Administrative denotes management or support tasks in health care organizations, such as patient registration, admission, and discharge. Strategic represents tasks performed by management teams in health care organizations, such as strategic planning decisions, human resources management, and performance evaluations. Research represents all activities that are related to medical research.

The user dimension relates to how-to knowledge and aggregates the possible user types of CCSs. This dimension differentiates between a patient who receives medical treatment at a health care organization, the medical staff (health care professionals as well as administrators), and the family members of the patient.

Service delivery device refers to how-to knowledge because this dimension represents the types of client devices used to access the CCS. A CCS with an independent characteristic allows users to access services using any computer or mobile device. Adapted specifies that a CCS is compatible with different types of devices but operates more efficiently on a certain group of devices (eg, mobile phones or tablets) via technical adaptation to those devices (eg, developing specialized applications for tablets or compressing data to accelerate data transfer for mobile phones). Specialized represents those CCSs that can be accessed by only 1 or several designated groups of devices, such as authorized tablet computers, workstations in health care organizations, or specific medical devices.

Finally, the patient data involvement dimension, which also relates to how-to knowledge, explains how patient-related data are used to deliver services. Internal indicates that a CCS uses patient data that are internally available to the health care organization for IT service delivery. External refers to a situation in which a CCS uses patient data collected from external sources, such as outside medical professionals or the patients themselves. No involvement indicates that a CCS does not have access to patient data and thus does not use such data in IT service delivery.

### Classification and Evaluation

After completing all taxonomy development iterations, we classified all 50 CCSs that we identified during stage 1. Multimedia Appendix 4 presents the final classification results. In this section, we provide an example of how our taxonomy can be used to classify CCSs for health care organizations. This example examines a hospital decision support system for bed-patient assignments (see C22, Multimedia Appendix 1). Because this CCS addresses patient administration and assists hospital leadership in measuring and benchmarking hospital operations, it supports both administrative and strategic tasks. The CCS is delivered in the form of a software application and is hosted in a public cloud environment. The targeted cloud advantage is scalability because the hospital benefits from CC’s computing resources to analyze large quantities of data based on complex mathematical models. The CCS does not operate in real time (not real time). It is used by medical staff and is not device-specific (independent). Finally, the patient data processed by the CCS are internal.

Our taxonomy fulfills all predefined ending conditions after 14 development iterations. In particular, the fulfillment of 5 subjective ending conditions indicates high sufficiency of the taxonomy. We summarized these subjective ending conditions and provide a justification for the fulfillment of each condition in Multimedia Appendix 5. Notably, the subjective ending conditions describe the essential features of the derived taxonomy.

## Discussion

### Principal Findings

#### Specific Meanings of Cloud Computing for Health Care and Research Opportunities

By observing the taxonomy, which includes the classification results of CCSs for health care organizations, we obtained specific implications of CCSs for health care.

**Table 3:** Specificities of cloud computing for health care.

Number	Specificity	Previous understanding	Type
1	CC^a^ relies on SaaS^b^	PaaS^c^ and IaaS^d^ in general are as relevant as SaaS	Type 1^e^
2	CC increases data security and interoperability	Low data security and interoperability as CC’s downside	Type 1
3	If any, CC only brings economic benefits in the long term	Reduced costs by using CC in general	Type 1
4	CC focuses on clinical tasks	Health IT^f^ traditionally supports more management areas	Type 2^g^
5	CC supports patient-centeredness	Health IT products are traditionally heavily physician-centered	Type 2
6	CC increases service mobility and flexibility	Health IT traditionally suffers from inflexible service access	Type 2
7	CC facilitates collaboration in clinical areas	Insufficient capabilities of traditional health IT to support collaboration	Type 2

^a^CC: cloud computing.

^b^SaaS: software as a service.

^c^PaaS: platofrm as a service.

^d^IaaS: infrastructure as a service.

^e^The specificity challenges what we have learned about CC in a general context.

^f^IT: information technology.

^g^The specificity challenges what we have learned about traditional health IT.

**Figure 3:**
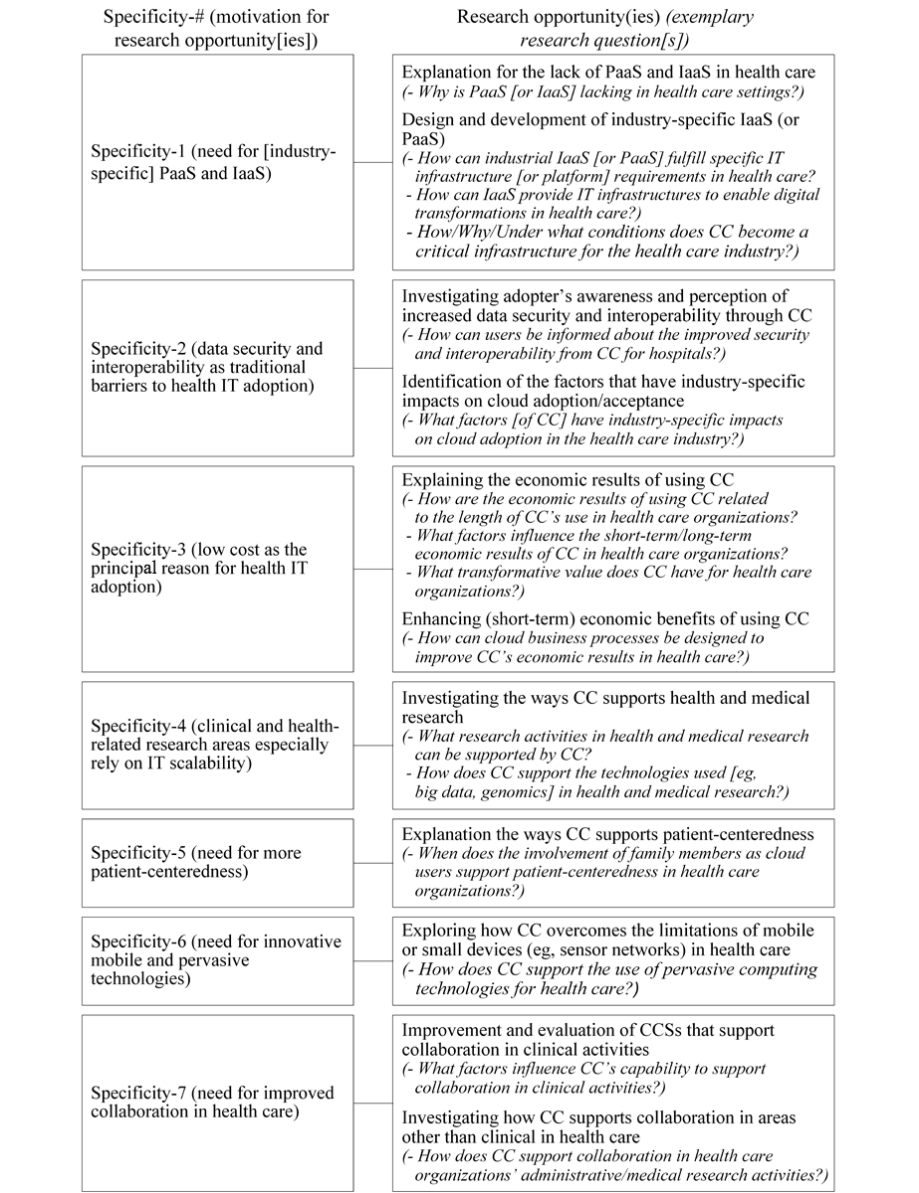
Research opportunities for cloud computing in health care. CC: cloud computing, CCS: cloud computing service, IaaS: infrastructure as a service, IT: information technology, PaaS: platform as a service.

As demonstrated in Table 3, these implications offer 2 types of challenges to our previous understanding of CC in health care: they challenge what we have learned about CC in a general context (type 1) and in published traditional health IT studies (type 2). We employed the term “specificities” to summarize these implications, thereby highlighting the specific meanings of CC for health care. More importantly, as shown in Figure 3, the summarized specificities suggest research opportunities with exemplary research questions, facilitating future research about this relevant phenomenon in health IT.

#### Specificity 1: Cloud Computing in Health Care Relies on Software as a Service

Previous studies show that in a common context, PaaS and IaaS are as relevant as SaaS in the cloud market [40]; however, this result is challenged by CC in the context of health care (type 1). We found that 92% (46/50) of the CSSs deliver services in the form of SaaS (dimension service form). The identified research articles and the interviewees even applied the term “X as a service,” such as “hospital information system as a service” [41] or “documentation as a service” (i17), to emphasize the importance of such CCSs, although by their nature they belong to SaaS. This is possibly because health care organizations expect to exploit the advantages of SaaS to the greatest extent and in a timely manner.

For hospitals, cloud almost only means software as a service because many hospitals want to use (them as) off-the-shelf products. ...SaaS products that support medical areas are especially welcome because hospitals always expect to get immediate improvement from the cloud in their core business.

Interviewee i03

The lack of PaaS and IaaS in health care organizations indicates an insufficient state of CC in health care, which was confirmed by several interviewees (i07-i08, i10, i17-i19). For PaaS, our taxonomy shows only one CCS (C06), although several interviewees noted the urgent need for industry-specific PaaS.

We want to develop our own SaaS, but there is just no specific PaaS for health care organizations. General PaaS are not enough.

Interviewee i07

The need for PaaS in health care is not only because PaaS in general provides ready-to-use technical support for programmers but also because it has the potential to provide solutions to effectively fulfill industry-specific IT requirements. This is, for example, explained by an interviewee who was involved in developing a CCS for a hospital.

There were so many complex things we had to consider for hospitals. We kept wasting time on unnecessary meetings to find technical solutions. I dreamt of having a PaaS that could support us. ...Of course, there is more. ...Compliance is also a main topic. Hospitals ask over and over again whether our software is compliant with this or that. ...Example HIPAA: If the PaaS we use is compliant with HIPAA, then we can tell them: Yes, our software is HIPAA-compliant.

Interviewee i17

Further industry-specific IT requirements that can potentially be supported by a health care PaaS—constant demand on cutting-edge technologies, high health IT agility (to meet changing medical requirements), the need for different domain-specific medical data structures, and support for industrial joint implementation activities (eg, between government and hospital)—were also mentioned by the interviewees.

For IaaS, previous research studies [42] and our interviewees both emphasized the strategic meaning (i08) of IT infrastructure (ie, critical information infrastructure) for the health care industry and consequently the extremely high importance of IaaS (i20) for health care organizations. We identified only a limited number of IaaS (n=3) used for general administration of health care organizations (C28, C37) or data storage (C38), which hardly fulfills all health care organization IT infrastructure requirements.

Future research could focus on exploring the lack of PaaS and IaaS for health care. As revealed by our interview data, there is a particular need for research studies that systematically investigate specific requirements for health care that cannot be covered by PaaS and IaaS in a common context and thus a need to design and develop industry-specific PaaS and IaaS.

#### Specificity 2: Cloud Computing Brings More Data Security and Interoperability to Health Care

Previous studies have raised concerns about security and privacy as the Achilles heel of CC [43], which are main barriers for the adoption of health IT artifacts [44,45]. These concerns might be more severe for public clouds, whose infrastructures are accessible by many different users [46]. However, the dimension deployment model indicates that more than half of the investigated CCSs are based on public clouds, especially given that almost all of these CCSs involved patient data (dimension: patient data involvement) that were sensitive and entailed security or privacy issues. To this end, providing a high level of data security was regarded as a targeted cloud advantage in 10 of the identified CCSs, of which 6 were deployed on public clouds. This challenges our understanding of CC in a general context (type 1). Additionally, interoperability may also impede the adoption of CC in a general context [47]. For health care, however, our taxonomy demonstrates that increased interoperability is a benefit of CC. Security and interoperability are traditionally the most intractable challenges in health IT, and industry standards concerning IT security and interoperability in health care are evolving [9]. Cloud providers can devote resources to the implementation of industry standards or best practices that many hospitals cannot afford [4]. CC can thereby address security and interoperability issues in a more effective manner, which was confirmed by the interviewed experts (i03-i04, i06-i07, i10, i13-i14, i16-i18, i21).

CC is safe. The problem is how to make people believe that.

Interviewee i13

Data security, interoperability...these are pluses. Speaking of data security, using paper is also not safe, if you insist on saying a cloud is not safe.

Interviewee i21

As highlighted in Figure 3, future research could investigate the role of security and interoperability in cloud adoption studies and focus on the adopter’s awareness or perception of increased data security and interoperability from CC in health care settings. Moreover, researchers could focus on exploring the factors (such as security and interoperability) that have industry-specific impacts on cloud adoption in health care, in contrast to a general context.

#### Specificity 3: Cloud Computing Brings Economic Benefits to Health Care Organizations, if Any, Only in the Long Term

It is surprising that CC offered economic advantages (cost efficiency) for only 11 of the 50 CCSs. In a general context, the use of CC is heavily motivated by short-term economic interests [48]. Research relying on this general understanding of CC claimed the low costs were the principle advantage of CC in health care [4]. Our research challenges the understanding of CC in a general context (type 1) by revealing that when using CCSs, many health care organizations frequently must transfer large volumes of data to and from the cloud (eg, medical images [49]). This can cause data transfer bottlenecks due to the obsolete (network) infrastructures currently in place at many health care organizations—a typical industry-specific IT issue (i02, i08, i15). Thus, CC might still require signiﬁcant short-term investments in health care organizations’ network resources, internet bandwidth, or other relevant infrastructures. It is therefore not surprising that the interviewees were not convinced of the potential financial advantages of using CC in health care (i01-i05, i07, i10, i17). They (i01-i02, i10) even noted that additional expenses for CC, such as consulting fees, could increase health care organizations’ expenses. However, our interviewees reported that in the long term, CC will reduce their general IT maintenance work (i02, i24) and help them avoid possible IT reinvestments (i22). Future research could therefore focus on (re)examining and explaining the economic results of using CCSs in health care organizations. Moreover, researchers could focus on CC business processes or investment strategies in health care settings that enhance the short-term benefits for health care organizations.

#### Specificity 4: Cloud Computing Mainly Focuses on Clinical Tasks (by Leveraging High Scalability)

We recognize that most of the identified CCSs (36 of 50) support clinical tasks in health care organizations (dimension: supported task). This observation challenges previous studies about traditional health IT (type 2), which have concluded that health care organizations primarily focus on the use of IT applications for administrative, strategic, or financial functions rather than clinical activities [50]. These findings reflect an urgent need to use CC to remedy the deficiencies of traditional health IT in the context of health care organizations’ clinical activities, as revealed by our literature review [51].

In clinical practice, even ordinary data analysis occasionally overwhelms traditional health IT with large volumes of data and complex analytical algorithms.

Interviewee i16

CC can address this problem with highly scalable IT resources and is therefore considered a “powerful weapon for IT tasks in the clinical area” (Interviewee i03).

This viewpoint is supported by our taxonomy, as more than 70% (23/32) of the CCSs possessed high scalability as one of their advantages (dimension: targeted cloud advantage), with a focus on clinical areas. For research opportunities, we suggest researchers concentrate on CC that supports research tasks in health care because both the literature [52] and our interviewees (eg, i18) reveal that research activities in health care depend even more on highly scalable IT resources to address large amounts of data, which is currently managed only in a small number of identified CCSs (n=6).

#### Specificity 5: Cloud Computing Supports Patient-Centeredness

A conservative but still well-recognized view of health IT is that medical staff are the main users of health IT applications [53,54], and many existing health IT applications are heavily physician-centered. However, the evidence from our taxonomy challenges this view (type 2) and implies a high potential of CC to realize patient-centeredness—a promising future direction for health IT [55]. Regarding the user dimension, we noticed that 8 identified CCSs included patients as their users, which is a premise of patient-centered health IT services. Among them, 7 CCSs were patient-centered (C05, C07, C10, C26, C29, C32, C34), as they possessed 3 essential attributes of patient-centered health IT: patient-focused, patient-active, and patient-empowered [56]. Additionally, several interviewees (i02, i07-i08, i11) noted that CC innovatively involves patient family members to realize patient-centeredness, as did 2 identified CCSs (C26, C29). An interviewee, whose hospital deploys a medical appointment CCS for patients, had this to say:

Seniors, the disabled, or someone who doesn’t like technologies also needs to use appointment services, so we decided to involve their relatives. ...Although we have to have more users and processes now, I believe CC can offer the necessary computer resources. It’s a good thing, and I think this might be a reason to have more CCSs.

Interviewee i02

We even have some patients who don’t use the Internet at all. Their children could help them...only in this way can we ensure that each patient truly benefits from our services.

Interviewee i08

Despite the potential of CC to support patient-centeredness, only a limited number of patient-centered CCSs were identified in this study. Future research could therefore focus on examining how CC supports patient-centeredness and on designing further CCSs that support it.

#### Specificity 6: Cloud Computing Increases Service Mobility and Flexibility

We found that 42% (21/50) of the identified CCSs adapt themselves to or are specialized for certain devices for service delivery (dimension: service delivery device). For CCSs that support clinical tasks, this rate is even higher (16/36, 45%). In general, a barrier impeding the use of health IT is the alteration of users’ traditional workflow paradigm [57]. For health IT that supports clinical functions, physicians who are forced to adapt health care delivery processes to technologies are often unwilling to use it. Our taxonomy reveals that almost 80% (16/21) of the CCSs that were adapted to user devices, such as mobile phones and tablet PCs or other specialized medical devices, targeted service ubiquity (dimension: targeted cloud advantage) and thus the mobility and flexibility of IT service delivery (type 2). Existing health IT research concluded that these devices are inherently subjected to limited computing capacity and are criticized as unsuitable for complex tasks, such as clinical work [58]. However, our research shows that more than one-third (8/21) of the CCSs that were adapted to user devices enjoyed the benefit of resource scalability (dimension: targeted cloud advantage). Thus, as emphasized by our interviewees, CC can effectively “offset the [traditional] limitations of mobile devices or other small devices. It can increase the use of innovative devices in health care” (Interviewee i07). Future research could explore how CC overcomes the limitations of mobile or small devices in health care, which is a relevant but underinvestigated topic in health IT [58].

#### Specificity 7: Cloud Computing Facilitates Collaboration in Clinical Settings.

Our taxonomy demonstrates that most of the CCSs (46/50) involved the use of patient data (dimension: patient data involvement). One major expected purpose of involving patient data in health IT is to employ the data as a means to link users or systems in different clinical areas and thereby facilitate their collaboration [59]. However, research generally highlights a lack of sufficient health IT applications that support collaboration [60]. Our taxonomy challenges this (type 2) and reveals that CC has the potential to address this issue, as 21 of the 46 CCSs (that involve patient data and support clinical areas) possessed shareability or interoperability as an advantage (dimension: targeted cloud advantage) and had improved collaboration between users or systems as one of their main purposes. However, these CCSs are not without limitations. Only a small fraction of these CCSs (6/21) involved patient data from external sources (dimension: patient data involved). Including patient data from different sources is the basis of collaboration in clinical activities [51]. Our interviewees (i02, i05, i08, i11, i15) noted that including patient data from external sources (eg, external medical professionals or patients themselves) is relevant for improving collaboration in clinical processes because “no hospitals can depend only on themselves. They need continual cooperation with, at least, patients” (Interviewee i02).

The interviewees remarked that CCSs in health care organizations that have a collaboration purpose mostly focus on internal data exchanges (which was also revealed by our taxonomy), although they believed that CC has the potential to also facilitate collaboration with external parties. The timeliness dimension is another indicator for collaboration because it addresses how intensively data exchanges occur. However, for the 21 CCSs that supported clinical areas and possessed the shareability or interoperability characteristics, we found that only 8 enabled real-time data exchanges. Real time is crucial for effective data exchanges and the resulting collaboration in clinical processes (i05-i06, i08, i11, i18).

Collaboration [based on data exchanges] should not only take place but also in a real-time manner. A delay of important data for even a few minutes could be fatal for clinical activities.

Interviewee i08

Future research should therefore strive to improve CCSs for collaboration in clinical activities due to the currently (still) insufficient state of CCSs (as well as general health IT [51,60]) for supporting collaboration. Moreover, researchers could also investigate how CC supports collaboration in areas other than clinical settings in health care.

### Contributions

For health IT research, our contributions are threefold. First, we suggest a taxonomy that structures the knowledge of CCSs (ie, CCS properties) for health care organizations. In particular, our taxonomy targets principle and how-to knowledge to systematically conceptualize the concept of CC for health care settings. Unlike previous research that heavily relied on CC literature from common contexts or on traditional understandings of health IT, our study analyzed CC’s industry-specific properties not only from the health IT literature but also from practice. Thus, the derived dimensions and characteristics of the taxonomy highlight the aspects of CC that are most relevant to health care. We thereby contribute to closing the gap between an insufficient conceptual understanding of CC and the actual phenomenon in practice for health care. Second, our taxonomy suggests 7 specificities that subvert and thus challenge our previous understanding of CC in a general context or of traditional health IT. These specificities advance the understanding of CC in health care. Third, we derived concrete research opportunities for health IT (see Multimedia Appendix 6 for a summary). As presented at the beginning, health IT researchers have been interested in the development of single CC applications or data security topics. For both topics, we provide suggestions that guide future research (eg, to focus on developing CCSs that enable collaboration in health care) or even create new opportunities and directions (eg, to focus on inherently increased, instead of decreased, IT security in health care by using CC). In addition, we noticed that research topics on CC are by nature broad and diverse, which should not be limited to the development of CC applications and IT security, as in current health care settings, but can include more areas such as its business perspective [61,62], its adoption (by organizations) [63,64], user awareness and acceptance [65,66], and its certification [67-69]. The proposed research directions in this study are a step toward facilitating research on CC in health care settings.

For health IT practice, the derived taxonomy can be applied to investigate CCSs for health care organizations on 2 different levels. On a macro level, the classification of available CCSs in a certain health IT market using the taxonomy can serve as an indicator of the current state of these CCSs. Cloud providers or policy makers could, for example, suggest new CCSs that address possible market gaps (eg, PaaS for hospitals). On a micro level, health care organizations could apply the taxonomy to understand an individual CCS. In particular, by combining the characteristics from the dimensions that a CCS possesses, health care organizations could specify each CCS’s profile as demonstrated, for example, by the hospital decision support system for bed-patient assignments, as referred to in the Results section. By finding matches as well as mismatches between the CCS’s profile and their own organizational needs, health care organizations could screen and identify CCSs that would be useful to them and thereby increase the meaningful use of CC.

### Limitations and Conclusions

A main limitation of this research is that our data focused on health care organizations that are hospitals and clinics, as implied by the literature review search string and by the interview questions. This is because hospitals and clinics are not only the backbone of the health care industry [70] but also representative IT consumers in health care [71]. We therefore expected that a taxonomy derived from hospitals and clinics would provide more generally valid insights into CC for health care settings. Research that focuses on CC in more specific health care settings (eg, nursing homes) could employ our taxonomy as a starting point. We suggest that such research use the proposed dimensions and characteristics as a checklist to investigate CC. If required, adjustments along the taxonomy’s dimensions and/or characteristics can be easily carried out [32], resulting in more specific taxonomies that are useful for certain health care settings. Future research should also broaden the perspective on the topic of CC to cover further health care settings by using, for example, more general search strings for literature reviews (eg, including terms such as “health IT” and “eHealth”) or by designing interview topics that cover CCSs in other health care areas.

Our work relied on data from 24 expert interviews, which does not necessarily guarantee that all CCSs for health care organizations from practice were discovered. However, the selection of our interviewees ensured a wide spectrum of knowledge about CC in health care in Asia, Western Europe, and the United States, which represent the main CC health care markets. Future research could also include niche CC markets to further verify and improve our taxonomy.

Although the term “cloud computing” has existed since 2007, the phenomenon of CC in health care remains in its infancy and calls for research on this phenomenon have emerged [4,25]. By relying on perspectives from a taxonomy for CCSs for health care organizations, we provide a solid conceptual cornerstone for research about CC in health care; moreover, the suggested specificities of CC for health care and the related future research opportunities will serve as a valuable roadmap.

## Appendix

Multimedia Appendix 1 Overview of identified cloud computing services.

Multimedia Appendix 2 Overview of interview questions.

Multimedia Appendix 3 Taxonomy development iterations.

Multimedia Appendix 4 Taxonomy of cloud computing services for health care organizations.

Multimedia Appendix 5 Taxonomy’s fulfillment of the subjective ending conditions.

Multimedia Appendix 6 Future research directions.

## Abbreviations

CC: cloud computing
CCS: cloud computing service
IaaS: infrastructure as a service
IT: information technology
PaaS: platform as a service
RQ: research question
SaaS: software as a service
