# Supplementary material for: Rethinking the Meaning of Cloud Computing for Health Care: A Taxonomic Perspective and Future Research Directions
Source: J Med Internet Res. 2018 Jul 11;20(7):e10041. doi: 10.2196/10041 (PMC6060303; doi:10.2196/10041)
Supplement: Multimedia Appendix 1 [file jmir_v20i7e10041_app1.pdf]

Multimedia Appendix 1: Overview of Identified Cloud Computing Services

ID	Short description [source(s)][a]
C01	C01 is a cloud-based clinical decision support system that is used for early recognition of a certain disease by means of analysis of the patient [1]
C02	C02 is a cloud computing service (CCS) that is used for the recognition of and treatment for an infant disease. Based on analyses of patient data, C02 supports treatment scheduling and coordinates treatment processes across different units within a hospital [2]
C03	By storing, managing and exchanging a data index in the cloud, C03 enables users to search for and share patient data stored in different information systems from different hospitals [3]
C04	C04 is a cloud-based electronic health record (EHR) system that supports a hospital in collecting health-related information by using mobile devices in rural areas [4]
C05	C05 is an outpatient monitoring system for pregnant women [5]
C06	C06 is cloud-based platform that supports the development and deployment of health-related IT services in a hospital. In particular, C06 can be used to secure, capture, store and consume sensitive healthcare data [6]
C07	C07 is a cloud-based EHR system that supports the management, storage, access, and sharing of patient data within a hospital [7]
C08	C08 is a real-time remote consultation system for emergency medical services based on patients' electrocardiography. C08 is used to process, visualize and share patient data and support decision making [8]
C09	C09 is used to support a complex treatment process within a hospital. The system monitors and guides different stages of the process [9]
C10	C10 is a patient monitoring system for chronic disease treatment. It monitors patients' vital signs and enables remote medical consultation [10]
C11	C11 is a picture archiving and communication system that is used to store and process medical images in a hospital [11]
C12	C12 is a cloud-based EHR system owned by a large-sized city hospital that supports outside clinics and doctors in rural areas [12]
C13	C13 is a cloud-based EHR system that supports offsite remote access of internal patient data using mobile devices [13]
C14	C14 is a cloud-based system for processing and storing medical data in a hospital [14]
C15	Based on analyses of patient data, C15 is a remote patient monitoring system for critical care [15]
C16	C16 is a monitoring system for patients in cardiac rehabilitation [16]
C17	C17 is a cloud-based picture archiving and communication system for sharing medical images between different users and terminals [17]
C18	C18 is a service for accessing and sharing patient data across different hospitals for disaster response [18]

C1 9	C19 is a service owned by a hospital that allows outside medical professionals to order laboratory tests and access test results [19]

Multimedia Appendix 1: Continued

ID	Short description [source(s)][a]
C20	C20 is an ERP system that supports emergency medical services by providing integrated patient data [20]
C21	C21 is a cloud system for the transmission of the results of electrocardiograms [21]
C22	C22 is a hospital decision support system that employs complex mathematical models to periodically recommend bed-patient assignments [22]
C23	C23 is a virtual desktop service that is shared by a group of hospitals [23]
C24	C24 is a virtual desktop solution for all areas in a hospital [24]
C25	C25 supports clinical decisions for a complex disease based on analyses of patient data [25]
C26	C26 is an IT service that enables patients and their relatives to book and manage medical appointments at a hospital. C26 also allows patients to view laboratory reports related to their appointments and upload their own image files (e.g., medical image data from other hospitals) to support diagnoses [i01, i07]
C27	C27 is a CCS provided for physicians in a hospital that allows them to view and share patient information with each other using authorized mobile devices [i01]
C28	C28 is an IT service that provides IT infrastructure for IT platforms and IT applications for a hospital's administrative departments [i02, i12]
C29	C29 is a medical appointment management tool that enables patients to book medical appointments in a hospital. C29 also allows patients to view laboratory reports related to their medical appointments [i02, i04, i10]
C30	C30 is an IT service for physicians in a general hospital that enables its users to directly view and share medical images on authorized tablet computers [i03]
C31	C31 is a web service that is used to support radiology information systems and provides enterprise-wide image processing (e.g., 3D/4D visualization) across clinical care areas in a hospital [i03]
C32	C32 is a web-based CCS that enables patients to book medical appointments for certain top-rated hospitals in Shanghai [i04, i08]
C33	C33 is a cloud-based platform provided for a group of small-sized hospitals in a district of a Chinese city. C08 enables the processing and sharing of patients' electronic medical records and thus facilitates collaboration within these hospitals [i05]
C34	C34 is a web application that provides remote real-time medical consultation services for patients. C34 enables real-time voice and video communications between physicians and patients. C09 employs sensors and other specialized medical devices to collect patients' vital signs, such as

	blood pressure and heart rate [i05, i11]

Multimedia Appendix 1: Continued

ID	Short description [source(s)][a]
C35	C35 is an internet-based cloud solution for hospital information systems in dental hospitals that integrates all standard applications and information systems for a common dental hospital [i06]
C36	C36 is a cloud-based virtual desktop solution deployed in a general hospital. C11 covers all applications available in the hospital [i08, i09, i22]
C37	C37 is a cloud service that provides IT infrastructure for whole hospital areas [i12]
C38	C38 is a cloud-based service that provides IT storage infrastructure for medical image archiving systems in hospitals. [i13, i14, i16]
C39	C39 is a cloud service used by hospitals to assess their key performance [i13, i14]
C40	C40 is a cloud service for a community of hospitals used to share medical image data, exchange opinions on medical images, and transfer patient data [i15]
C41	C41 is a browser-based system used to document and monitor oncological clinical studies in hospitals [i17, i19]
C42	C42 is a decision support system for sample selection and patient recruitment for oncological clinical studies in hospitals [i17, i19]
C43	C43 is a web-based solution that can be used to centrally store and process medical image data for a hospital. C43 also provides e-learning based on its image data [i18]
C44	C44 is a web-based IT service that enables data-sharing for research or administration purposes between different partner hospitals [i20, i22, i23]
C45	C45 is a data storage service for long-term archiving of clinical image data in hospitals [i20, i24]
C46	C46 is web-based service used to view, search and share clinical reference information for medical research [i21]
C47	C47 is a web-based IT solution for employee recruitment and staff training management in a hospital [i22]
C48	C48 is a cloud service used for food selection and purchase in a hospital [i23]
C49	C49 is an IT service used to manage all construction projects in a hospital. Users can view the status and technical details of all on-going projects and communicate with building contractors [i23]
C50	C50 is a cloud-based solution that supports a hospital's daily office business, such as word and table processing, email functions, and schedule management [i24]

Note:

a. For data sources from expert interviews (labelled with [i#]) cf. Table 1 in the paper)

References

1. Amland RC, Hahn-Cover KE. Clinical Decision Support for Early Recognition of Sepsis. American Journal of Medical Quality 2014;31(2):103-110. PMID:25385815
2. Arnold RW, Jacob J, Matrix Z. A Cloud-Based Electronic Medical Record for Scheduling, Tracking, and Documenting Examinations and Treatment of Retinopathy of Prematurity. Journal of Pediatric Ophthalmology & Strabismus 2012;49(6):342-346. PMID:22800793
3. Bhaskaran S, Suryanarayana G, Basu A, Joseph R. Cloud-Enabled Search for Disparate Healthcare Data: a Case Study. 2013 IEEE International Conference on Cloud Computing in Emerging Markets (CCEM); 2013 Oct 16-18; Bangalore, India. IEEE; 2013. doi:10.1109/CCEM.2013.6684431
4. Boonchieng E, Boonchieng W, Senaratana W, Singkaew J. Development of mHealth for Public Health Information Collection, with GIS, Using Private Cloud: a Case Study of Saraphi District, Chiang Mai, Thailand. 2014 International Computer Science and Engineering Conference (ICSEC); 2014 Jul 30-Aug 1; Khon Kaen, Thailand. IEEE; 2014. doi:10.1109/ICSEC.2014.6978221
5. Crişan-Vida M, Serban A, Ghihor-Izdrăilă I, Mirea A, Stoicu-Tivadar L. User Friendly IT Services for Monitoring and Prevention During Pregnancy. Studies in Health Technology and Informatics 2014;202:241-244. PMID: 25000061
6. Fan L, Buchanan W, Thummler C, Lo O, Khedim A, Uthmani O, Lawson A, Bell D. DACAR Platform for eHealth Services Cloud. 2011 IEEE 4th International Conference on Cloud Computing (CLOUD); 2011 Jul 4-9; Washington, DC, USA. IEEE; 2011. doi:10.1109/CLOUD.2011.31
7. Fernández-Cardeñosa G, de la Torre-Díez, I, López-Coronado M, Rodrigues, JJPC. Analysis of Cloud-Based Solutions on EHRs Systems in Different Scenarios. Journal of Medical Systems 2012;36(6):3777-3782. PMID:22492177
8. Hsieh J, Hsu M. A Cloud Computing Based 12-Lead ECG Telemedicine Service. BMC Medical Informatics and Decision Making 2012;12(77). PMID:22838382
9. Hu N, Lee C, Hou MC, Chen Y. A Cloud System for Mobile Medical Services of Traditional Chinese Medicine. Journal of Medical Systems 2013;37(6):1-13. PMID:24081559
10. Jeong S, Kim S, Kim D, Youn C, Kim Y. A Personalized Healthcare System for Chronic Disease Care in Home-Hospital Cloud Environments. 2013 International Conference on ICT Convergence (ICTC); 2013 Oct 14-16; Jeju Island, Korea. IEEE; 2013. doi: 10.1109/ICTC.2013.6675375
11. Karthikeyan N, Sukanesh R. Cloud Based Emergency Health Care Information Service in India. Journal of Medical Systems 2012;36(6):4031-4036. PMID:22865161

12. Lin C, Abdul SS, Clinciu DL, Scholl J, Jin X, Lu H, Chen SS, Iqbal U, Heineck MJ, Li Y. Empowering Village Doctors and Enhancing Rural Healthcare Using Cloud Computing in a Rural Area of Mainland China. Computer Methods and Programs in Biomedicine 2014;113(2):585-592. PMID:24296075
13. Lomotey RK, Deters R. Supporting N-Screen Medical Data Access in mHealth. 2013 IEEE International Conference on Healthcare Informatics (ICHI); 2013 Sep 9-11; Philadelphia, PA, USA. IEEE; 2013. doi: 10.1109/ICHI.2013.38
14. Maratt JD, Srinivasan RC, Dahl WJ, Schilling PL, Urquhart AG. Cloud-Based Preoperative Planning for Total Hip Arthroplasty: a Study of Accuracy, Efficiency, and Compliance. Orthopedics 2012;35(8):682-686. PMID:22868590
15. McGregor C. A Cloud Computing Framework for Real-Time Rural and Remote Service of Critical Care. 2011 24th International Symposium on Computer-Based Medical Systems (CBMS); 2011 Jun 27-30; Bristol, UK. IEEE; 2011. doi:10.1109/CBMS.2011.5999037
16. Mesa I, Sanchez E, Toro C, Diaz J, Artetxe A, Graña M, Guijarro F, Martinez C, Jimenez JM, Rajasekharan S, Alarcon JA, de Mauro A. Design and Development of a Mobile Cardiac Rehabilitation System. Cybernetics and Systems 2014;45(2):92-108. doi:10.1080/01969722.2014.874804
17. Mezger U, Jendrewski C, Bartels M. Navigation in Surgery. Langenbeck's Archives of Surgery 2013;398(4):501-514. PMID:23430289
18. Nagata T, Halamka J, Himeno S, Himeno A, Kennochi H, Hashizume M. Using a Cloud-Based Electronic Health Record During Disaster Response: a Case Study in Fukushima, March 2011. Prehospital and Disaster Medicine 2013;28(4):383-387. PMID:23731545
19. Pantanowitz L, LaBranche W, Lareau W. Stepwise Approach to Establishing Multiple Outreach Laboratory Information System-Electronic Medical Record Interfaces. Journal of Pathology Informatics 2010;1(5). PMID:20805958
20. Poulymenopoulou M, Malamateniou F, Vassilacopoulos G. E-EPR: a Workflow-Based Electronic Emergency Patient Record. Personal and Ubiquitous Computing 2014;18(1):91-100. doi:10.1007/s00779-012-0620-4
21. Takeuchi I, Fujita H, Ohe K, Imaki R, Sato N, Soma K, Niwano S, Izumi T. Initial Experience of Mobile Cloud ECG System Contributing to the Shortening of Door to Balloon Time in an Acute Myocardial Infarction Patient. International Heart Journal 2013;54(1):45-47. PMID: 23428924
22. Thomas BG, Bollapragada S, Akbay K, Toledano D, Katlic P, Dulgeroglu O, Yang D. Automated Bed Assignments in a Complex and Dynamic Hospital Environment. Interfaces 2013;43(5):435-448. doi:10.1287/inte.2013.0701
23. Yao Q, Han X, Ma X, Xue Y, Chen Y, Li J. Cloud-Based Hospital Information System as a Service for Grassroots Healthcare Institutions. Journal of Medical Systems 2014;38:104. PMID:25015761
24. Yoo S, Kim S, Kim T, Kim JS, Baek R, Suh CS, Chung CY, Hwang H. Implementation Issues of Virtual Desktop Infrastructure and Its Case Study for a Physician's Round at Seoul National University Bundang Hospital. Healthcare Informatics Research 2012;18(4):259-565. PMID:23346476

25. Yu H, Lai H, Chen K, Chou H, Wu J, Dorjgochoo S, Mendjargal A, Altangerel E, Tien Y, Hsueh C, Lai F. A Sharable Cloud-Based Pancreaticoduodenectomy Collaborative Database for Physicians: Emphasis on Security and Clinical Rule Supporting. Computer Methods and Programs in Biomedicine 2013;111(2):488-497. PMID:23706526
