# Supplementary material for: Rethinking the Meaning of Cloud Computing for Health Care: A Taxonomic Perspective and Future Research Directions
Source: J Med Internet Res. 2018 Jul 11;20(7):e10041. doi: 10.2196/10041 (PMC6060303; doi:10.2196/10041)
Supplement: Multimedia Appendix 2 [file jmir_v20i7e10041_app2.pdf]

Multimedia Appendix 2: Overview of Interview Questions

#	Question	Purpose
1	Where do you work? Please describe your working position and your scope of duties or your working activities in your organization.	To identify interviewee's scope of duties and thus validate her eligibility for the interview
2	How many years of work experience do you have in your line of work?	To determine interviewee's professional experience
3	How many employees do you have in your organization?	To obtain information about the organization size
4	What do you understand about cloud computing in health care organizations?	To verify expert's qualifications and obtain data about the concept of cloud computing in health care organizations
5	What do cloud computing mean to health care organizations?	To verify expert's qualifications and obtain data about the concept of cloud computing in health care organizations
6	Is your hospital/clinic currently using any cloud computing services? Is your organization providing any cloud services for hospitals/clinics? What are these cloud services?	To address concrete cloud services for health care organizations, in which the interviewee is involved
7	Do you know of any other cloud computing services being used by hospitals or clinics that you are familiar with? What are these cloud computing services?	To address further cloud services for health care organizations that the interviewee is familiar with
8	For each of the cloud computing services you mentioned, what concrete services does it provide?	To identify the purpose of each concrete cloud computing service
9	For each of the cloud computing services you mentioned, please describe how it works and supports hospitals.	To address the basic functions of each concrete cloud computing service
10	For each of the cloud computing services you mentioned, what concrete features or characteristics does it possess?	To address the features of each concrete cloud computing service
11	For each of the cloud computing services, is there any further information about it? Do you have any further comments?	To address further possibly useful information about each cloud computing service
