# Supplementary material for: Rethinking the Meaning of Cloud Computing for Health Care: A Taxonomic Perspective and Future Research Directions
Source: J Med Internet Res. 2018 Jul 11;20(7):e10041. doi: 10.2196/10041 (PMC6060303; doi:10.2196/10041)
Supplement: Multimedia Appendix 3 [file jmir_v20i7e10041_app3.pdf]

Multimedia Appendix 3: Taxonomy Development Iterations

Iteration	Approach	Data sources[a]
1	deductive	[1–4] i09; i16; i20; i21
2	deductive	[5–9] i12; i13; i24
3	inductive	C16; C25; C33; C39; C49; C50
4	deductive	[10–13] i04; i11; i18; i23
5	inductive	C01; C04; C07; C22; C23; C24; C40;
6	inductive	C14; C29; C34; C35; C46; C42
7	deductive	[14–17] i05; i08; i10; i22
8	inductive	C02; C05; C12; C15; C37; C41
9	deductive	[18–22] i02; i07; i14
10	inductive	C17; C20; C26; C28;C30; C44
11	inductive	C08; C11; C21; C31; C45; C48
12	deductive	[23,24] i01; i03; i06; i15; i17; i19;
13	inductive	C06; C09; C13; C32; C38; C43
14	inductive	C03; C10; C18; C19; C27; C36; C47

Note:

a. For data sources labelled with ‘i#’ cf. Table 1 in the paper. For data sources labelled with ‘C#’ cf. Multimedia Appendix 1

References

1. Banerjee A, Agrawal P, Rajkumar R. Design of a Cloud Based Emergency Healthcare Service Model. International Journal of Applied Engineering Research 2013;8(19):2261-2264.
2. Li-Ming W, Hui W, Hong W. Hospital Digital Library Based on Cloud Computing. 2014 IEEE Workshop on Advanced Research and Technology in Industry Applications (WARTIA); 2014 Sep 29-30; Ottawa, ON, Canada. IEEE; 2014. doi: 10.1109/WARTIA.2014.6976406
3. Low C, Chen YH. Criteria for the Evaluation of a Cloud-Based Hospital Information System Outsourcing Provider. Journal of Medical Systems 2012;36(6):3543-3553 PMID:22366976
4. Mendelson DS, Erickson BJ, Choy G. Image Sharing: Evolving Solutions in the Age of Interoperability. Journal of the American College of Radiology 2014;11(12):1260-1269 PMID:25467903
5. He C, Jin X, Zhao Z, Xiang T. A Cloud Computing Solution for Hospital Information System. 2010 IEEE International Conference on Intelligent Computing and Intelligent Systems (ICIS 2010); 2010 Oct 29-31; Xiamen, China. IEEE;2010. doi: 10.1109/ICICISYS.2010.5658278
6. Kanagaraj G, Sumathi AC. Proposal of an Open-Source Cloud Computing System for Exchanging Medical Images of a Hospital Information System. 2011 3rd International Conference on Trendz in Information Sciences and Computing (TISC); 2011 Dec 8-9; Chennai, India. IEEE; 2011. doi: 10.1109/TISC.2011.6169102
7. San OY, Husain W. Maternity Data Management Utilizing Cloud Computing. 2014 International Conference on Computer and Information Sciences (ICCOINS); 2014 Jun 3-5; Kuala Lumpur, Malaysia. IEEE; 2014. doi:10.1109/ICCOINS.2014.6868422
8. Serban A, Crişan-Vida M, Stoicu-Tivadar L. Data and Knowledge in Medical Distributed Applications. Studies in Health Technology and Informatics 2014;197:41-45. PMID:24743075
9. Stylianides N, Gjermundrod H, Dikaiakos M, Kyprianou T. Intensive Care Cloud: Exploiting Cloud Infrastructures for Near Real-Time Vital Sign Analysis in Intensive Care Medicine. 2012 IEEE 12th International Conference on Bioinformatics & Bioengineering (BIBE); 2012 Nov 11-13; Larnaca, Cyprus. IEEE; 2012. doi: 10.1109/BIBE.2012.6399697
10. Paules CA, Fardoun HM, Alghazzawi DM, Oadah M. KAU E-Health Mobile System. Proceedings of the 13th International Conference on Interacción Persona-Ordenador. 2012 Oct 3-5; Elche, Spain. New York: ACM; 2012. doi:10.1145/2379636.2379664
11. Lee S, Song JH, Kim IK. CDA Generation and Integration for Health Information Exchange Based on Cloud Computing System. IEEE Transactions on Services Computing 2014;9(2):241-249. doi:10.1109/TSC.2014.2363654
12. Ratnam KA, Dominic, PDD, Ramayah T. A Structural Equation Modeling Approach for the Adoption of Cloud Computing to Enhance the Malaysian Healthcare Sector. Journal of Medical Systems 2014;38:82 PMID:24957398

13. Vadaliya P, Laroia M, Kar G. InterCARE: A Cloud Computing Patient Care System. Proceedings of the India HCI 2014 Conference on Human Computer Interaction. 2014 Oct 3-5; New Delhi, India. New York: ACM; 2014. doi:10.1145/2676702.2676714
14. Boiron P, Dussaux V. Healthcare Software as a Service: The Greater Paris Region Program Experience -- the So-called "Région Sans Film" Program. 2011 16th IEEE International Enterprise Distributed Object Computing Conference Workshops (EDOCW); 2011 Aug 29-Sep 2; Helsinki, Finland. IEEE; 2011. doi: 10.1109/EDOCW.2011.38
15. Dixon BE, Simonaitis L, Goldberg HS, Paterno MD, Schaeffer M, Hongsermeier T, Wright A, Middleton B. A Pilot Study of Distributed Knowledge Management and Clinical Decision Support in the Cloud. Artificial Intelligence in Medicine 2013;59(1):45-53 PMID:23545327
16. Hsieh J, Li A, Yang C. Mobile, Cloud, and Big Data Computing: Contributions, Challenges, and New Directions in Telecardiology. International Journal of Environmental Research and Public Health 2013;10(11):6131-6153 PMID:24232290
17. Kharat A, Safvi A, Thind SS, Singh A. Cloud Computing for Radiologists. Indian Journal of Radiology and Imaging 2012;22(3):150-154 PMID:23599560
18. Rajkumar R. P2P Cloud Architecture for Rural Health Center: Monitoring and Evaluation. International Journal of Applied Engineering Research 2013;8(19):2293-2294.
19. Basu S, Karp AH, Li J, Pruyne J, Rolia J, Singhal S, Suermondt J, Swaminathan R. Fusion: Managing Healthcare Records at Cloud Scale. Computer 2012;45(11):42-49. doi:10.1109/MC.2012.291
20. Krestin GP, Grenier PA, Hricak H, Jackson VP, Khong PL, Miller JC, Muellner A, Schwaiger M, Thrall JH. Integrated Diagnostics: Proceedings from the 9th Biennial Symposium of the International Society for Strategic Studies in Radiology. European Radiology 2012;22(11):2283-2294 PMID:22699871
21. Schoenhagen P, Zimmermann M, Falkner J. Advanced 3-D Analysis, Client-Server Systems, and Cloud Computing-Integration of Cardiovascular Imaging Data into Clinical Workflows of Transcatheter Aortic Valve Replacement. Cardiovascular Diagnosis and Therapy 2013;3(2):80-92 PMID:24282750
22. Zhou F, Cheng F, Wei L, Fang Z. Cloud Service Platform - Hospital Information Exchange (HIX). 2011 IEEE 8th International Conference on e-Business Engineering (ICEBE); 2011 Oct 19-21; Beijing, China. IEEE; 2011. doi:10.1109/ICEBE.2011.35
23. Kagadis GC, Kloukinas C, Moore K, Philbin J, Papadimitroulas P, Alexakos C, Nagy PG, Visvikis D, Hendee WR. Cloud Computing in Medical Imaging. Medical Physics 2013;40(7):70901 PMID:23822402
24. Rajkumar R, Chackravatula N. Dynamic Integration of Mobile JXTA with Cloud Computing for Emergency Rural Public Health Care. Osong Public Health and Research Perspectives 2013;4(5):255-264 PMID:24298441
