# Supplementary material for: Rethinking the Meaning of Cloud Computing for Health Care: A Taxonomic Perspective and Future Research Directions
Source: J Med Internet Res. 2018 Jul 11;20(7):e10041. doi: 10.2196/10041 (PMC6060303; doi:10.2196/10041)
Supplement: Multimedia Appendix 4 [file jmir_v20i7e10041_app4.pdf]

Multimedia Appendix 4: Taxonomy of Cloud Computing Services (CCSs) for Health Care Organizations

CCS[a]	ST				SF			DM			TCA							T		U			SDD			PDI		
	cl	ad	sg	re	sw	pf	if	pb	pr	co	sc	el	ub	ce	sh	io	se	rt	nr	pa	ms	fm	ip	at	sp	in	ex	ni
C01	√				√			√			√					√			√		√		√			√		
C02	√	√			√			√			√		√						√		√		√			√		
C03	√				√			√			√				√	√			√		√		√				√	
C04		√			√			√					√		√				√		√				√		√	
C05	√				√			√			√		√					√		√	√				√		√	
C06	√					√			√		√			√			√		√		√		√			√		
C07	√				√			√			√		√		√				√	√	√			√		√		
C08	√		√		√			√					√		√		√	√			√			√			√	
C09	√				√				√							√			√		√			√		√		
C10	√				√				√				√		√			√		√	√				√		√	
C11	√				√			√			√			√					√		√		√			√		
C12	√				√				√		√				√				√		√		√			√		
C13	√				√				√				√		√				√		√				√	√		
C14	√				√			√			√			√					√		√		√			√		
C15	√			√	√			√			√		√			√		√			√		√				√	
C16	√				√			√			√		√					√		√	√				√		√	
C17	√				√			√			√				√				√		√				√	√		
C18	√				√				√		√		√		√				√		√			√		√		
C19		√			√				√		√					√			√		√		√				√	
C20	√				√			√					√		√		√	√			√			√		√		
C21	√				√			√					√		√		√	√			√			√		√		
C22		√	√		√			√			√								√		√		√			√		
C23	√				√					√	√				√				√		√		√			√		
C24	√	√	√	√	√				√			√	√	√					√		√		√			√		
C25	√				√					√	√				√		√		√		√		√			√		
C26	√	√			√				√				√						√	√		√			√		√	
C27	√				√				√				√		√				√		√				√	√		
C28		√					√		√			√						√			√		√			√		
C29		√			√				√				√						√	√		√			√		√	
C30	√				√				√				√		√				√		√				√	√		
C31	√				√			√			√			√					√		√		√			√		
C32		√			√			√	√		√							√		√			√			√		
C33	√				√					√	√				√		√		√		√		√				√	
C34	√				√			√			√		√					√		√	√				√		√	
C35	√	√	√	√	√			√			√			√					√		√		√			√		
C36	√	√	√	√	√				√			√	√	√					√		√		√			√		
C37	√	√	√	√			√	√			√	√		√				√			√		√			√		
C38	√						√		√		√			√			√	√			√		√			√		
C39			√		√			√			√								√		√		√			√		

Multimedia Appendix 4: Continued

CCS[a]	ST				SF			DM			TCA							T		U			SDD			PDI		
	cl	ad	sg	re	sw	pf	if	pb	pr	co	sc	el	ub	ce	sh	io	se	rt	nr	pa	ms	fm	ip	at	sp	in	ex	ni
C40	√				√					√					√			√			√		√				√	
C41				√	√			√			√					√	√	√			√			√		√		
C42		√			√			√								√	√	√			√			√			√	
C43	√		√		√				√		√				√			√			√		√			√		
C44		√		√	√			√			√				√			√			√		√					√
C45	√				√			√			√			√			√		√		√		√			√		
C46				√	√			√			√				√				√		√		√				√	
C47			√		√				√					√				√			√		√					√
C48		√			√			√			√								√		√		√					√
C49			√		√			√			√				√			√			√			√				√
C50	√	√	√	√	√			√					√		√			√			√			√		√		
**Hits (N)**	36	16	11	9	46	1	3	29	18	4	32	4	20	11	22	7	10	20	30	8	47	2	29	10	11	31	15	4

Abbreviations:
ad (Administrative); at (Adapted); CCS (Cloud computing service); ce (Cost efficiency); cl (Clinical); co (Community); DM (Deployment model); el (Elasticity); ex (External); fm (Family member); if (Infrastructure); in (Internal); io (Interoperability); ip (Independent); ms (Medical staff); ni (No involvement); nr (Not real-time); pa (Patient); pb (Public); PDI (Patient data involvement); pf (Platform); pr (Private); re (Research); rt (Real-time); sc (Scalability); SDD (Service delivery device); se (Security); SF (Service form); sg (Strategic); sh (Shareability); sp (Specialized); ST (Supported task); sw (Software); T (Timeliness); TCA (Targeted cloud advantage); U (User); ub (Ubiquity)

Note:

a. For the descriptions of the cloud computing services (labelled with C#) cf. Multimedia Appendix 1
