# Supplementary material for: Rethinking the Meaning of Cloud Computing for Health Care: A Taxonomic Perspective and Future Research Directions
Source: J Med Internet Res. 2018 Jul 11;20(7):e10041. doi: 10.2196/10041 (PMC6060303; doi:10.2196/10041)
Supplement: Multimedia Appendix 5 [file jmir_v20i7e10041_app5.pdf]

Multimedia Appendix 5: Taxonomy's Fulfilment of the Subjective Ending Conditions

Condition[a]	Definition	Justification for the fulfilment
Concise	The number of dimensions in a taxonomy should fall in the range of seven plus or minus two, such that the taxonomy is not overwhelming.	Our taxonomy is composed of eight dimensions.
Robust	A taxonomy should contain enough dimensions and characteristics to adequately differentiate the objects of interest.	In our taxonomy, each dimension is a distinct facet of a cloud computing service (CCS) and contains two to seven characteristics. The taxonomy yields a large number of characteristic combinations to differentiate among CCSs.
Compre-hensive	A taxonomy should classify all known objects within the domain or include all dimensions of objects of interest.	Although a complete evaluation of all existing CCSs in health care is not guaranteed, we provide a long list of existing CCSs that can be classified using our taxonomy. Our taxonomy was developed on the basis of studies covering a broad spectrum of topics about cloud computing that are applicable to empirical data from the expert interviews. Thus, the taxonomy covers a wide range of perspectives related to its meta-characteristic.
Extendible	A useful taxonomy permits the inclusion of additional dimensions and characteristics within a dimension if new types of objects appear.	Additional dimensions and/or characteristics can be easily added to our taxonomy (for example, if new CCSs are developed that provide novel service models for hospitals, their corresponding characteristics can easily be added to the dimension service form).
Explanatory	It should be clear what the taxonomy's dimensions and characteristics explain about an object.	Guided by how-to knowledge and principle knowledge, our taxonomy explains how CCSs can be used by hospitals and why they support hospitals.

Note:
a. The subjective ending conditions are according to Nickerson et al. [1]

References

1. Nickerson RC, Varshney U, Muntermann J. A Method for Taxonomy Development and Its Application in Information Systems. European Journal of Information Systems 2013;22(3):336-359. doi:10.1057/ejis.2012.26
