# Supplementary material for: Rethinking the Meaning of Cloud Computing for Health Care: A Taxonomic Perspective and Future Research Directions
Source: J Med Internet Res. 2018 Jul 11;20(7):e10041. doi: 10.2196/10041 (PMC6060303; doi:10.2196/10041)
Supplement: Multimedia Appendix 6 [file jmir_v20i7e10041_app6.pdf]

Multimedia Appendix 6: Future Research Directions

Future research direction	Exemplary research questions
Explanation for the lack of IaaS and PaaS in health care	• Why is IaaS (or PaaS) lacking in health care settings?
Design and development of industry-specific IaaS (or PaaS)	• How can industrial IaaS (or PaaS) fulfill specific IT infrastructure (or platform) requirements in health care?<br>• How can IaaS provide IT infrastructures to enable digital transformations in health care?<br>• How/Why/Under what conditions does CC become a critical infrastructure for the health care industry?
Investigating adopter's awareness and perception of increased data security and interoperability through CC	• How can users be informed about the improved security and interoperability from CC for hospitals?
Identification of the factors that have industry-specific impacts on cloud adoption/acceptance	• What factors (of CC) have industry-specific impacts on cloud adoption in the health care industry?
Explaining the economic results of using CC	• How are the economic results of using CC related to the length of CC's use in health care organizations?<br>• What factors influence the short-term/long-term economic results of CC in health care organizations?<br>• What transformative value does CC have for health care organizations?
Enhancing (short-term) economic benefits of using CC	• How can cloud business processes be designed to improve CC's economic results in health care?
Investigating the ways CC supports care-related research	• What research activities in medical research can be supported by CC?<br>• How does CC support the technologies used (eg, big data) in medical research?
Explanation the ways CC supports patient-centeredness	• When does the involvement of family members as cloud users support patient-centeredness in health care organizations?
Exploring how CC overcomes the limitations of mobile or small devices (eg, sensor networks) in health care	• How does CC support the use of pervasive computing technologies for health care?
Improvement and evaluation of CCSs that support collaboration in clinical activities	• What factors influence CC's capability to support collaboration in clinical activities?
Investigating how CC supports collaboration in areas other than clinical in health care	• How does CC support collaboration in health care organizations' administrative/ medical research activities?
